# Cardiac lipid content is unresponsive to a physical activity training intervention in type 2 diabetic patients, despite improved ejection fraction

**DOI:** 10.1186/1475-2840-10-47

**Published:** 2011-05-26

**Authors:** Vera B Schrauwen-Hinderling, Ruth CR Meex, Matthijs KC Hesselink, Tineke van de Weijer, Tim Leiner, Michael Schär, Hildo J Lamb, Joachim E Wildberger, Jan FC Glatz, Patrick Schrauwen, M Eline Kooi

**Affiliations:** 1 NUTRIM, School of Nutrition, Toxicology and Metabolism, Maastricht University Medical Center, Maastricht, The Netherlands; 2 Department of Radiology, Maastricht University Medical Center, Maastricht, The Netherlands; 3 Department of Human Movement Sciences, Maastricht University Medical Center, Maastricht, The Netherlands; 4 Biology of Lipid Metabolism Laboratory, Department of Physiology, Monash University, Clayton, Victoria, Australia; 5 Department of Human Biology, Maastricht University Medical Center, Maastricht, The Netherlands; 6 Cardiovascular Research Institute, CARIM, Maastricht University Medical Center, Maastricht, The Netherlands; 7 Department of Radiology, Utrecht University Medical Center, Utrecht, The Netherlands; 8 Department of Radiology, Johns Hopkins University, Baltimore, Maryland, USA; 9 Philips Healthcare, Cleveland, Ohio, USA; 10 Department of Radiology, Leiden University Medical Center, Leiden, The Netherlands; 11 Department of Molecular Genetics, Maastricht University Medical Center, Maastricht, The Netherlands

**Keywords:** magnetic resonance spectroscopy, magnetic resonance imaging, ectopic fat, type 2 diabetes mellitus, exercise, cardiomyopathy, lipotoxicity

## Abstract

**Background:**

Increased cardiac lipid content has been associated with diabetic cardiomyopathy. We recently showed that cardiac lipid content is reduced after 12 weeks of physical activity training in healthy overweight subjects. The beneficial effect of exercise training on cardiovascular risk is well established and the decrease in cardiac lipid content with exercise training in healthy overweight subjects was accompanied by improved ejection fraction. It is yet unclear whether diabetic patients respond similarly to physical activity training and whether a lowered lipid content in the heart is necessary for improvements in cardiac function. Here, we investigated whether exercise training is able to lower cardiac lipid content and improve cardiac function in type 2 diabetic patients.

**Methods:**

Eleven overweight-to-obese male patients with type 2 diabetes mellitus (age: 58.4 ± 0.9 years, BMI: 29.9 ± 0.01 kg/m^2^) followed a 12-week training program (combination endurance/strength training, three sessions/week). Before and after training, maximal whole body oxygen uptake (VO2max) and insulin sensitivity (by hyperinsulinemic, euglycemic clamp) was determined. Systolic function was determined under resting conditions by CINE-MRI and cardiac lipid content in the septum of the heart by Proton Magnetic Resonance Spectroscopy.

**Results:**

VO_2_max increased (from 27.1 ± 1.5 to 30.1 ± 1.6 ml/min/kg, p = 0.001) and insulin sensitivity improved upon training (insulin stimulated glucose disposal (delta Rd of glucose) improved from 5.8 ± 1.9 to 10.3 ± 2.0 μmol/kg/min, p = 0.02. Left-ventricular ejection fraction improved after training (from 50.5 ± 2.0 to 55.6 ± 1.5%, p = 0.01) as well as cardiac index and cardiac output. Unexpectedly, cardiac lipid content in the septum remained unchanged (from 0.80 ± 0.22% to 0.95 ± 0.21%, p = 0.15).

**Conclusions:**

Twelve weeks of progressive endurance/strength training was effective in improving VO_2_max, insulin sensitivity and cardiac function in patients with type 2 diabetes mellitus. However, cardiac lipid content remained unchanged. These data suggest that a decrease in cardiac lipid content in type 2 diabetic patients is not a prerequisite for improvements in cardiac function.

**Trial registration:**

ISRCTN: ISRCTN43780395

## Background

The prevalence of heart failure continues to increase. The life time risk to develop heart failure is presently 20% [1] and type 2 diabetes mellitus independently increases the risk for developing heart failure [2]. In addition to the well-known diabetes-associated risk of coronary atherosclerosis, also metabolic changes and increased triglyceride deposition in the diabetic myocardium are likely to contribute to the development of compromised cardiac function in patients with type 2 diabetes.

While most fatty acids (FA) in the body are stored in adipose tissue, small but physiologically relevant amounts are stored in non-adipose tissues such as skeletal muscle, liver, pancreas and the heart. In the heart, this (ectopic) fat storage has been suggested to interfere with insulin signaling and to induce apoptosis, which may contribute to impaired cardiac function, a process coined cardiac "lipotoxicity". Thus, lean rodent models with targeted overexpression of genes involved in lipid delivery and synthesis in the myocardium show lipid accumulation-induced dilated cardiomyopathy and heart failure in the absence of obesity-related cardiovascular risk factors (such as hypertension or dyslipidemia) [3-5], indicating that lipid accumulation in the myocardium can cause cardiomyopathy directly. Moreover, treatments ameliorating cardiac lipid accumulation rescued the heart from dilated cardiomyopathy in rodents [6,7].

In humans, cardiac lipid accretion has been reported in obesity and even more so in type 2 diabetes [8-10] although human data is still limited due to the fact that cardiac tissue samples are not readily available. Only recently, non-invasive lipid quantification in the septum of the heart using magnetic resonance spectroscopy has been established [11,12]. With this methodology, several studies showed inverse relations between cardiac lipid content and systolic and diastolic function [9-11], suggesting that excessive lipid accumulation in the heart may indeed impair normal cardiac function and result in cardiomyopathy and contribute to the increased risk of heart failure in type 2 diabetic patients [13]. However, whether a decrease in cardiac lipid content is necessary for improvement of cardiac function is presently unknown.

In that respect, we recently showed that endurance training reduced cardiac lipid content in parallel with an improvement in left ventricular ejection fraction in healthy, obese subjects [14]. It is well known that physical exercise training improves insulin sensitivity and reduces cardiac risk factors also in diabetic subjects. Hence, both the American Diabetes Association and American Heart Association promote regular physical exercise to reduce morbidity in type 2 diabetic patients [15]. Whether exercise training is also able to reduce cardiac lipid content in type 2 diabetic patients is presently unknown, and is the aim of the present study.

## Methods

### Subjects

Eleven male type 2 diabetic patients were included in this study. Diabetes had to be diagnosed for at least one year before the start of the study (on average: 3.5 ± 1.1 years) and was confirmed during screening (based on fasting plasma glucose concentrations of 7.0 mmol/l or higher, in accordance with the definition of the American Diabetes Association, ADA); diabetes was well-controlled in all subjects (HbA1c = 7.1 ± 0.2%). Exclusion criteria were: systolic blood pressure > 160 mm Hg or diastolic blood pressure > 100 mm Hg and known cardiac disease. Subjects' characteristics are listed in table 1. None of the patients was using insulin therapy; only oral anti-diabetic agents were used (metformin only (n = 6), metformin in combination with SU derivatives (n = 2) or SU derivatives only (n = 2)). Lipid-lowering medication was used by seven patients (six patients used statins, one patient used fibrates), some patients used blood pressure lowering medication (n = 5, of which 2 beta blokkers) or blood diluents (n = 4). Patients continued their normal medication throughout the study, except for antidiabetic medication which was stopped one week before the determination of insulin sensitivity. The study was approved by the institutional medical ethical committee and written informed consent was obtained from all participants.

**Table 1:** Subjects' characteristics before and after the training intervention period of 12 weeks.

	before training	after training	p-value
age (y)	59.5 ± 0.9	-	-

BMI (kg/m^2^)	30.5 ± 1.4	30.4 ± 1.4	0.8

Fat percentage (%)	30.3 ± 2.1	29.1 ± 2.1	0.11

VO_2_max (ml/kg/min)	27.1 ± 1.5	30.1 ± 1.6	0.001*

delta Rd (μmol/kg/min)	5.8 ± 1.9	10.3 ± 2.0	0.02 *

glucose (mMol)	9.1 ± 0.6	9.3 ± 0.6	0.6

HbA1c (%)	7.1 ± 0.2	7.2 ± 0.3	0.5

FA (μmol/l)	478.4 ± 54.2	526.3 ± 71.3	0.2

Total cholesterol (mmol/l)	4.9 ± 0.2	4.6 ± 0.3	0.3

HDL cholesterol (mmol/l)	1.2 ± 0.1	1.2 ± 0.1	0.6

LDL cholesterol (mmol/l)	2.7 ± 0.2	2.6 ± 0.2	0.4

Triacylglycerols (mmol/l)	2.1 ± 0.2	1.9 ± 0.2	0.4

Stars indicate statistically significant changes.

### Study protocol

Body composition, maximal oxygen uptake, insulin sensitivity, cardiac function and fat content, and fasting plasma concentration of fatty acids (FA), triacylglycerols, glucose, HbA1c, and cholesterol were measured before and after a 12-week well-controlled exercise program, consisting of a combination of aerobic and resistance exercise with three training sessions per week, carried out in small groups of 4 subjects, supervised by a coach, see [14].

### Maximal oxygen uptake (whole body oxidative capacity)

A routine incremental cycling test was used to determine the maximal aerobic capacity as described earlier (Oxycon Beta, Mijnhardt, The Netherlands) [14].

### Insulin sensitivity

Insulin sensitivity was measured with a hyperinsulinemic (40 mU/m^2^/min) euglycemic clamp, see [14]. Patients were asked to keep a constant eating pattern and to refrain from intense physical exercise during the last three days before the clamp.

### Hydrostatic weighing

Hydrostatic weighing with simultaneous measurement of lung volume was used to determine whole body fat percentage in the morning in the fasted state. The equation of Siri [16] was used to calculate fat percentage, fat mass and fat-free mass.

### Blood sample analysis

Blood samples were collected in EDTA-containing tubes and immediately centrifuged at high speed. Plasma FA, glucose and glycosylated haemoglobin were measured as described previously [14]. Total cholesterol, HDL-cholesterol and triacylglycerol were analyzed in serum enzymatically as described previously [14].

### MRI

An electrocardiographically triggered balanced steady state free precession (bSSFP) cine sequence was used to acquire images in two- and four chamber views and to image the whole heart in the short-axis orientation during breath holds (Intera, 1.5T, Philips Healthcare, Best, The Netherlands). Slice thickness was 6 mm with a 4 mm gap (flip angle = 50°; shortest possible TR (3.3 ms) and TE (1.67 ms); field of view = 350 × 350 mm, reconstructed matrix = 256 × 256, number of heart phases = 24). Cine images were analyzed using dedicated software (CAAS, Pie Medical Imaging, Maastricht, The Netherlands) to determine left ventricular end-systolic and end-diastolic volume. From this, parameters of systolic function were calculated (ejection volume, cardiac output, cardiac index). For one patient, the MRI procedure had to be accelerated due to anxiety and another patient had problems holding his breath. From these two subjects, it was impossible to acquire enough slices to cover the whole left ventricle and systolic function could not be determined and is therefore reported for nine subjects.

### MRS

**Figure 1:**
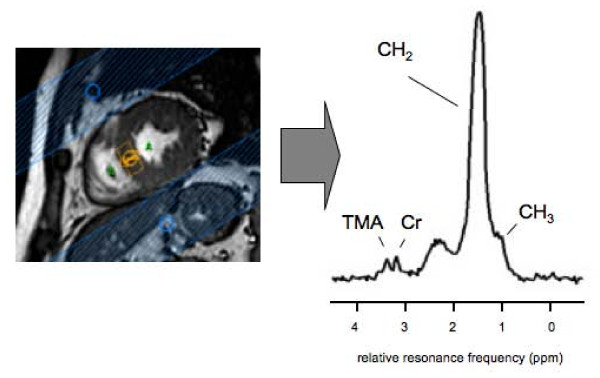
**A typical short axis view and a proton magnetic resonance spectrum, acquired from a volume of 10 × 20 × 30 mm^3 ^positioned in the septum of the heart**. The peaks of the CH_2 _and CH_3 _groups of the fatty acids can be discriminated and the peaks of Creatine (Cr) and trimethylammonium (TMA) are depicted in the figure.

Cardiac lipid content was determined *in vivo *by Magnetic Resonance Spectroscopy (MRS) as described previously [14]. Signal acquisition was restricted to a volume of interest of 6 cm^3 ^(10 mm × 20 mm × 30 mm) in the septum of the heart (PRESS sequence; spectral bandwidth = 1000 Hz; points acquired = 512; TE = 26 ms; TR = 4 s; n = 128 for lipid spectrum, n = 16 for reference water spectrum), signal acquisition was ECG-triggered to end-systole. Respiratory gating and tracking was performed with a pencil beam navigator placed on the diaphragm. A spectrum of the lipid metabolites was acquired with water suppression (CHESS). As internal reference, water signal intensity was determined in a spectrum with the water suppression pulses off-resonance. Overall acquisition time (imaging and spectroscopy) was 90 minutes. The coefficient of variation in repeated measurements was 11% on average. See figure 1 for a typical spectrum.

Peaks were fitted with Gaussian curves, imposing relative ratios of the amplitudes and line widths, using the AMARES algorithm [17] in jMRUI [18]. The results are displayed as ratios of the amplitudes of the CH_2 _peak relative to the water peak (uncorrected for T1 and T2 relaxation).

#### Statistics

All data are presented as mean ± SEM. Statistical analyses were performed using SPSS 16.0 for Mac (SPSS Inc., Chicago, IL). The effect of the intervention was determined by a two-sided, paired student t-test (considered significant if p < 0.05). Sample size (n = 11) was determined with β = 0.8 and α = 0.05, assuming࿠ a࿠ similar࿠ effect size as we found earlier

## Results

### Maximal oxygen uptake

The training program resulted in an improved maximal performance (from 200 ± 11 to 235 ± 13 Watt (p < 0.001). Accordingly, maximal whole body oxygen uptake improved with training (from 27.1 ± 1.5 to 30.1 ± 1.6 ml/min/kg (p = 0.001)).

### Body weight and whole body fat percentage

Body weight did not change with training (96.1 ± 4.0 and 95.6 ± 4.4 kg, pre- and post training, respectively, p = 0.8). Similarly, fat percentage, total body fat and fat-free mass remained unchanged (see table 1 for fat percentage).

### Plasma parameters

The training program did not affect fasting plasma glucose, HbA1c, FA and triacylglycerol plasma concentration. Total cholesterol concentrations, HDL and LDL cholesterol concentrations were unchanged after training (see table 1).

### Insulin sensitivity

Insulin sensitivity measured as insulin-stimulated glucose disposal (delta *R*d) during a hyperinsulinemic, euglycemic clamp improved significantly (from 5.8 ± 1.9 at baseline to 10.3 ± 2.0 mmol/kg/min after training, p = 0.02).

### Cardiac function and lipid content

**Figure 2:**
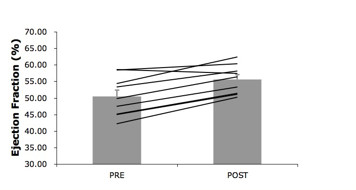
**Left ventricular ejection fraction improved with the training intervention (p = 0.01)**.

**Figure 3:**
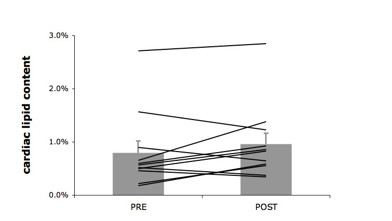
**Cardiac lipid content**. Cardiac lipid content before and after the training intervention. Cardiac lipid content is expressed as the relative intensity of the CH_2 _peak, compared to the unsuppressed water resonance. Cardiac lipid content is unchanged after the training intervention (p = 0.8).

Left ventricular end-diastolic volume was not affected by training (180.3 ± 8.7 to 179.0 ± 6.5 ml, p = 0.8) while end-systolic volume was reduced (from 89.6 ± 6.5 to 79.6 ± 4.5 ml, p = 0.004). The decreased end-systolic volume after training translates into improvements of similar magnitude (about 10%) of ejection fraction (from 50.5 ± 2.0 to 55.6 ± 1.5%, p = 0.01, see figure 2), cardiac index (from 2.87 ± 1.61 to 3.14 ± 1.08 l*min^-1^*m^-2^, p = 0.026) and cardiac output (from 5.8 ± 0.3 to 6.4 ± 0.2 ml/min, p = 0.04) after the training intervention. Strikingly, these adaptive changes occurred in the absence of changes in cardiac lipid content (0.80 ± 0.22% to 0.95 ± 0.21%, p = 0.15, as shown in figure 3.

Ejection fraction, cardiac index and cardiac output did not correlate with cardiac lipid content. Similarly, changes in cardiac function were not related to changes in cardiac lipid content.

## Discussion

The most important finding of the present study is that in this group of type 2 diabetic patients, a twelve-week training program, which increased aerobic capacity and insulin sensitivity resulted in an improvement of systolic function, while cardiac lipid content remained unchanged. These data suggest that a decrease in cardiac lipid content in type 2 diabetic patients is not a prerequisite for improvements in cardiac function. A limitation of the current study is the relatively small number of patients included.

Exercise training is well established as a powerful tool to reduce the risk for cardiovascular disease in type 2 diabetic patients [19-23]; however it is less clear whether training can actually improve cardiac function in this population. Data on the effect of training on systolic function in type 2 diabetic subjects is limited, with only one study reporting a positive effect [24] while others do not [25,26]. In diabetic animal models, however, the improvement in ejection fraction and fractional shortening with endurance training is well documented [27,28]. We previously reported improved ejection fraction in obese but otherwise healthy human subjects in response to exercise training [14]. Here, we show that a modest but significant improvement in ejection fraction can also occur in type 2 diabetic patients, in response to a relatively short period of training.

Rodent studies strongly support the concept that accumulation of triglycerides in cardiomyocytes causes cardiac lipotoxicity, impairing cardiac function. In addition, several human studies report negative correlations of triglyceride content with cardiac function as well [9,11]. Some [29], but not all [30] human studies report lowered cardiac lipid content upon thiazolidinediones. Recently, we reported that in healthy overweight-to-obese subjects, the training-induced improvement in systolic function is accompanied by a decrease in cardiac lipid, in line with the cardiac lipid hypothesis [14]. Intriguingly, though, exercise-induced improvement in cardiac contractile function was not accompanied by a decrease in lipid content in the patients with type 2 diabetes, rather cardiac lipid content tends to increase in this population, although not significantly. Why type 2 diabetic patients are unresponsive towards training-induced decrease in cardiac lipid content cannot be deduced from the present study. It is important to note that the cardiac lipid content in the present group of diabetic subjects is similar as reported in healthy overweight subjects of similar age and BMI [14]. However, when compared to healthy lean young men, cardiac lipid content is clearly elevated by ~300% in the present group of patients [31]. In other words, cardiac lipid content is elevated as expected based on BMI and age, however, it is not further elevated due to type 2 diabetes. This may be due to the relatively healthy patients investigated in the current study. Although diabetic, patients did not have any severe diabetes-related complications. Additionally, plasma concentrations of FFA and TG were also normal. However, although not elevated more severely than expected based on BMI and age at baseline, the cardiac lipid content was unresponsive to an exercise intervention in the diabetic subjects and interestingly, this differs from the response in healthy overweight subjects. It is yet unclear what is at the basis of this differential response, but it is well known that cardiac metabolism is altered in diabetes. For example, cardiac insulin resistance, decreased metabolic flexibility, mitochondrial dysfunction and excessive cardiac fat uptake and oxidation all have been associated with the diabetic state. Furthermore, a study in a diabetic animal model demonstrated decreased responsiveness to beta adrenergic stimulation in diabetic animals upon exercise training while beta adrenergic stimulation increased in control animals [32]. Future studies are needed to investigate whether these factors are contributing to the differential response in diabetic patients to exercise training resulting in the unchanged cardiac lipid content.

Furthermore, the present findings demonstrate that the training-induced improvement of systolic function can occur independently of changes in cardiac lipid content. Generally, it is believed that the beneficial effect of training on systolic- and diastolic function is based on alterations of the Ca^2+ ^regulatory systems involved in the excitation-contraction coupling and relaxation processes [33]. According to the cardiac lipotoxicity theory, there are potential mechanisms by which lipid accumulation may interact directly with excitation-contraction coupling. We here demonstrate that this is not pivotal: the improved function with exercise training can occur independently of changes in lipid content. Apparently, the improvement of cardiac function does not necessarily require decrease in cardiac lipids in type 2 diabetic subjects. Similarly, it has been shown that pioglitazone improves diastolic function in type 2 diabetic subjects without changes in cardiac lipid content [30].

## Conclusion

In conclusion, we report here that a twelve-week training program, which increased maximal whole body oxygen uptake and insulin sensitivity, resulted in an improvement in ejection fraction, while cardiac lipid content was unchanged. These results demonstrate that the training-induced improvement in systolic function does not necessarily require lowering of cardiac lipid availability. More studies are needed to get insight in the clinical relevance of cardiac lipid content and its relation with cardiac metabolism and diabetic cardiomyopathy.

## Competing interests

MS is employed by Philips Health Care, which does not pose a conflict of interest.

## Authors' contributions

VBSH: performing measurements, writing manuscript. RCM, TW: performing measurements, discussion of manuscript. TL: Discussion of data analysis and manuscript. MS, EK, HL: Helping to set up methodology, discussion of data and manuscript. JEW, JFCG: discussion of data and manuscript. PS, MKCH: experimental set-up, discussion of data and manuscript.
